# Supplementary material for: Fully exposed Pt clusters for efficient catalysis of multi-step hydrogenation reactions
Source: Nat Commun. 2024 Jun 7;15:4887. doi: 10.1038/s41467-024-49083-6 (PMC11161621; doi:10.1038/s41467-024-49083-6)
Supplement: Supplementary file 1 — Supplementary Information [file 41467_2024_49083_MOESM1_ESM.pdf]

Fully Exposed Pt Clusters for Efficient Catalysis of Multi-Step Hydrogenation Reactions

Yang Si[1, 2#], Yueyue Jiao[3, 4, 5#], Maolin Wang[6], Shengling Xiang[7], Jiangyong Diao[2*], Xiaowen Chen[1, 2], Jiawei Chen[1, 2], Yue Wang[8, 2], Dequan Xiao[9], Xiaodong Wen[3, 4], Ning Wang[7], Ding Ma[6*] and Hongyang Liu[1, 2*]

[1] School of Materials Science and Engineering, University of Science and Technology of China, Shenyang 110016, P. R. China.

[2] Shenyang National Laboratory for Materials Science, Institute of Metal Research, Chinese Academy of Sciences, Shenyang 110016, P. R. China.

[3] State Key Laboratory of Coal Conversion, Institute Coal Chemistry, Chinese Academy of Sciences, Taiyuan 030001, P. R. China.

[4] National Energy Center for Coal to Clean Fuel, Synfuels China Co., Ltd, Beijing 100871, P. R. China.

[5] The University of Chinese Academy of Sciences, Beijing 100049, P.R. China.

[6] Beijing National Laboratory for Molecular Sciences, New Cornerstone Science Laboratory, College of Chemistry and Molecular Engineering, Peking University, Beijing, China.

[7] Department of Physics and Center for Quantum Materials, Hong Kong University of Science and Technology, Kowloon, Hong Kong SAR, P. R. China.

[8] Department of Chemistry, Liaoning University, Shenyang, Liaoning 110036, China.

66 Chongshan Road.

[9] Center for Integrative Materials Discovery, Department of Chemistry and Chemical Engineering, University of New Haven, West Haven, Connecticut 06516, United States.

[#] These authors contributed equally to this work.

Corresponding Author:

*Jiangyong Diao (jydiao@imr.ac.cn), *Ding Ma (dma@pku.edu.cn) and *Hongyang Liu (liuhy@imr.ac.cn)

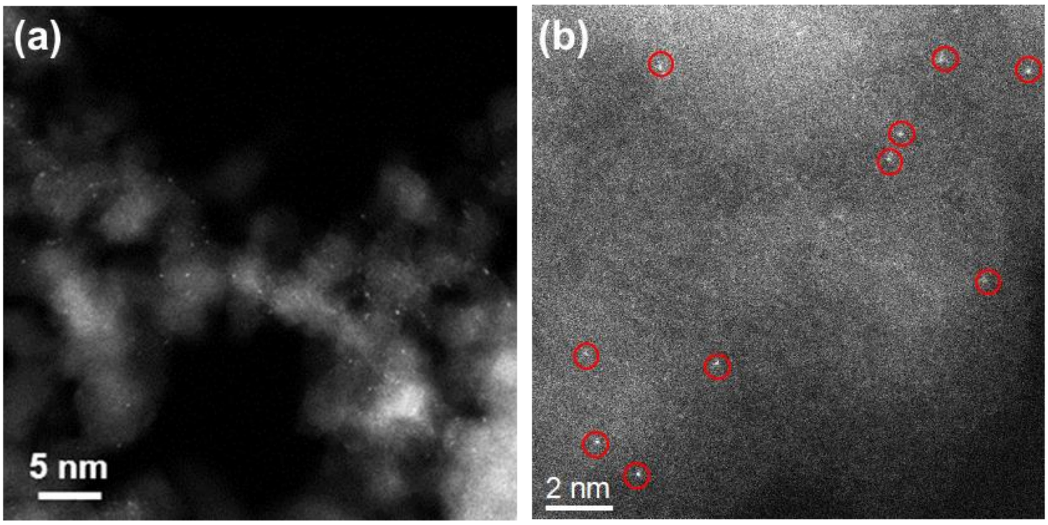

Supplementary Figure 1. AC-HAADF-STEM images. (**a**) low magnification images and (**b**) high magnification images of atomically dispersed Pt_1/ND@G.

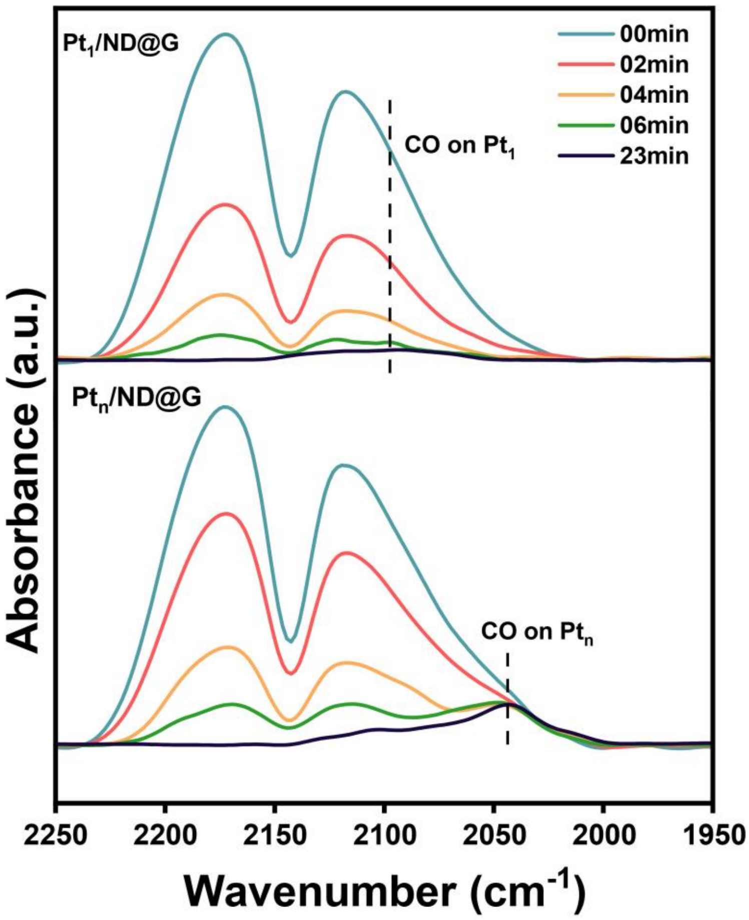

Supplementary Figure 2. *In situ* DRIFTS of CO adsorption. DRIFTS spectra of Pt_n/ND@G and Pt_1/ND@G

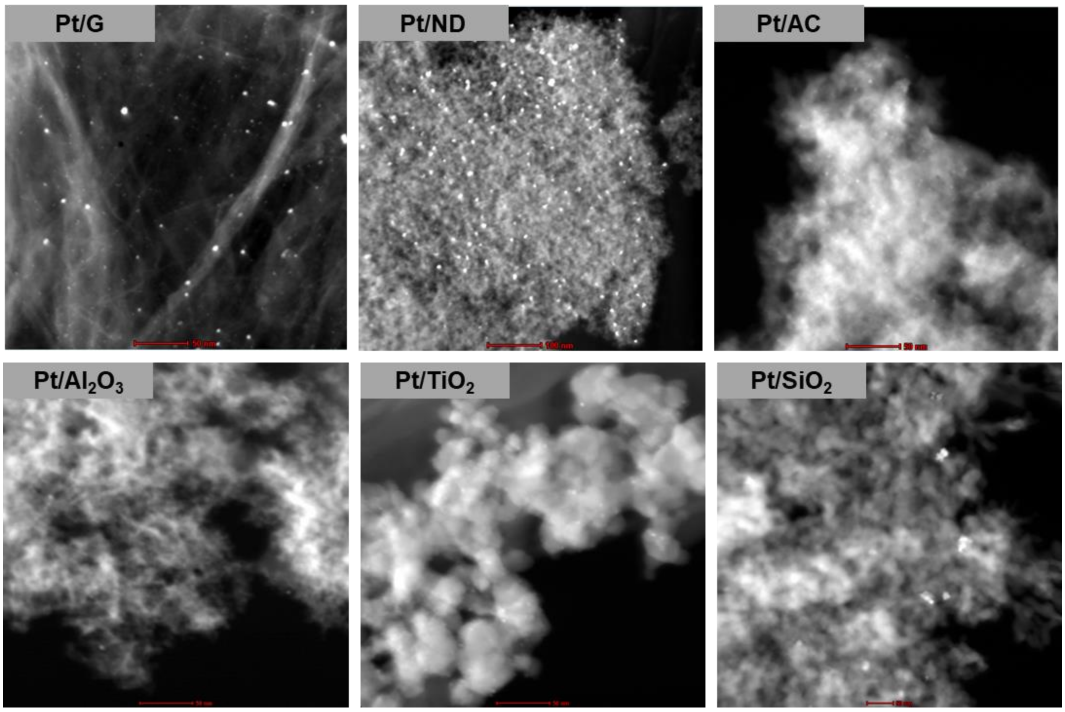

Supplementary Figure 3. STEM images. Images of Pt/G, Pt/ND, Pt/AC, Pt/Al_2O_3, Pt/TiO_2, Pt/SiO_2.

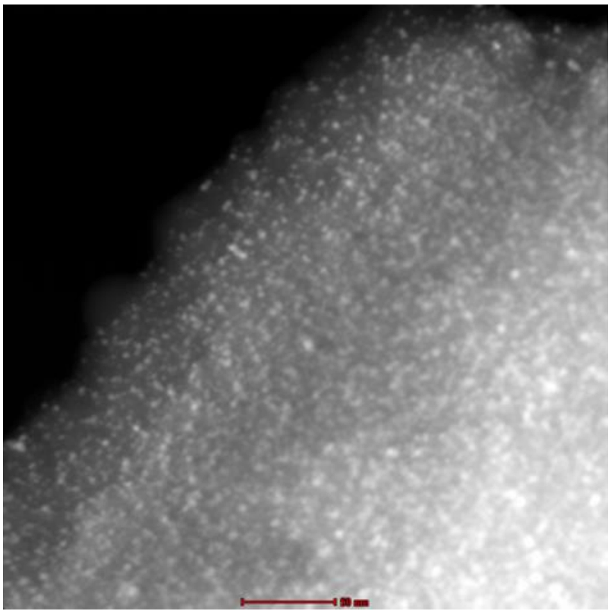

Supplementary Figure 4. STEM image. Image of 5wt% Pt/C.

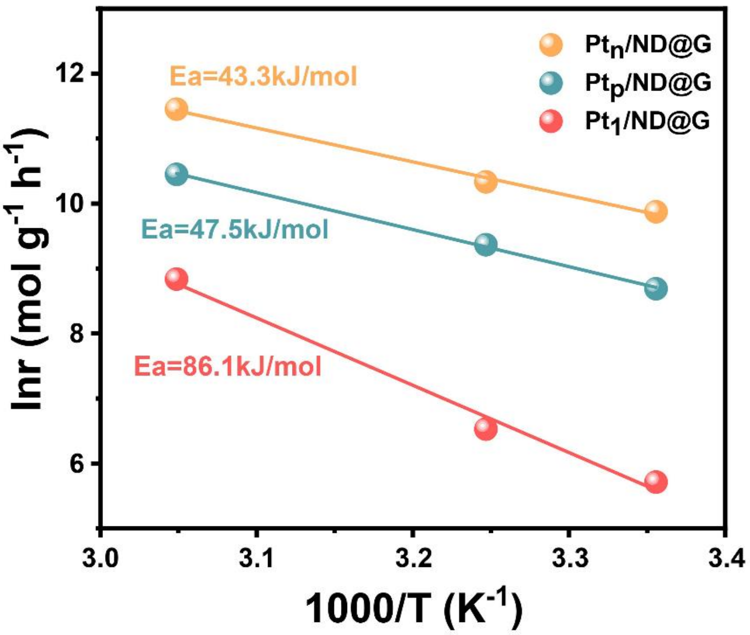

Supplementary Figure 5. Apparent activation energies. Calculated apparent activation energies through Arrhenius plots of Pt_1/ND@G, Pt_n/ND@G and Pt_p/ND@G for 2,4-DNT hydrogenation.

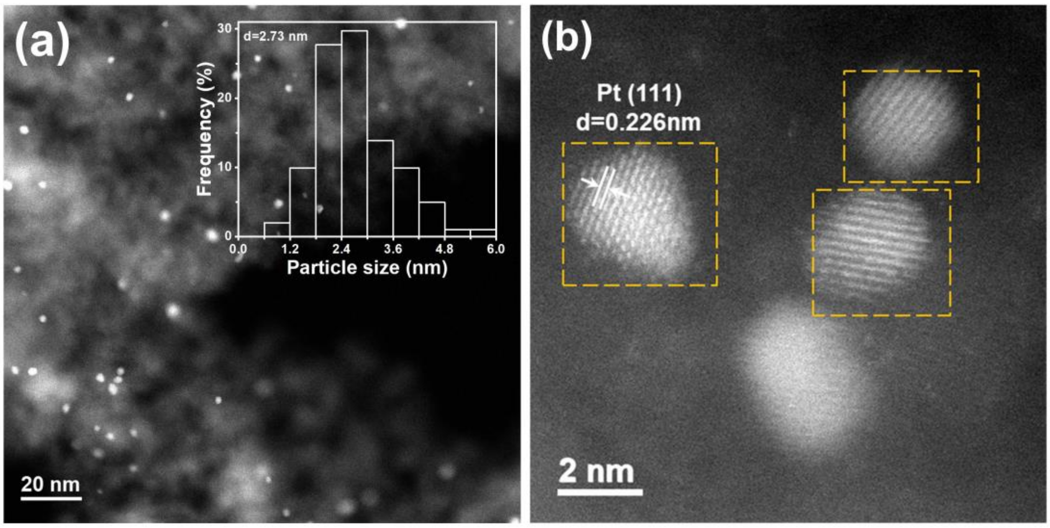

Supplementary Figure 6. AC-HAADF-STEM images. (**a**) low magnification images and (**b**) high magnification images of Pt_p/ND@G.

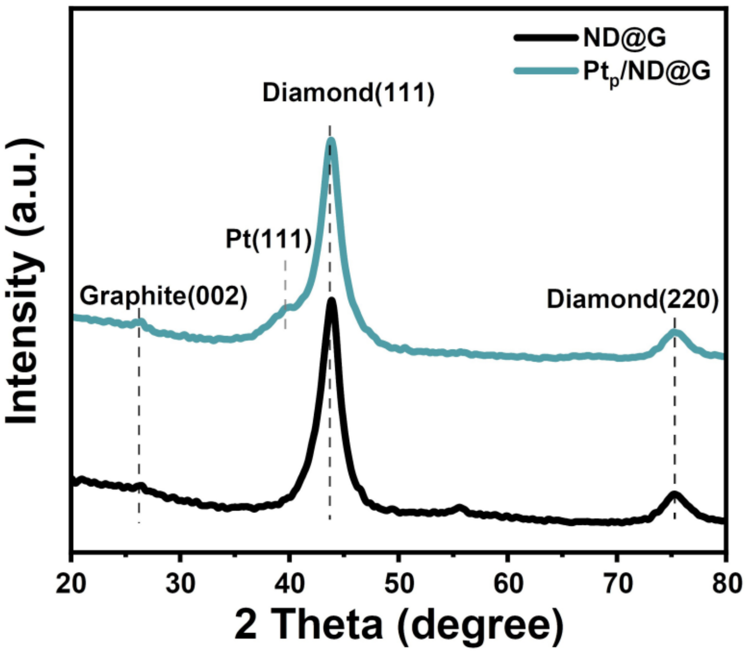

Supplementary Figure 7. XRD patterns. XRD of Pt$_p$/ND@G.

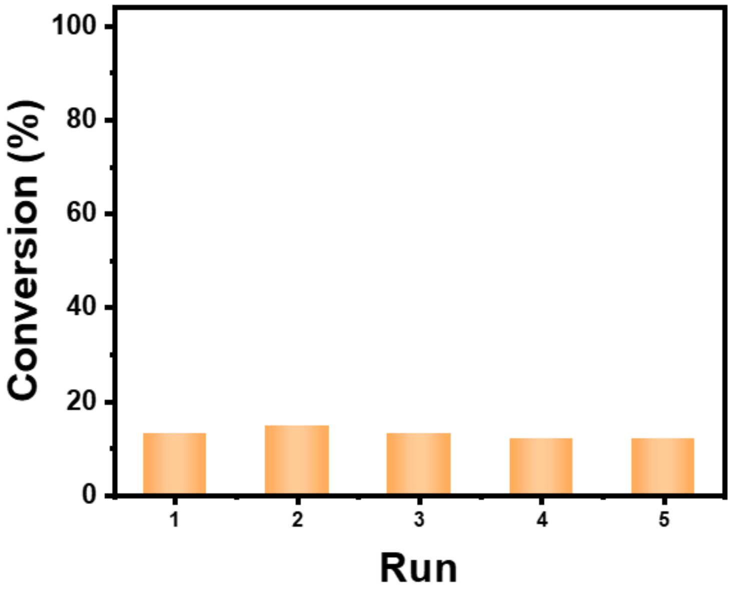

Supplementary Figure 8. Recycling test. Catalyst recycling test on Pt_n/ND@G.

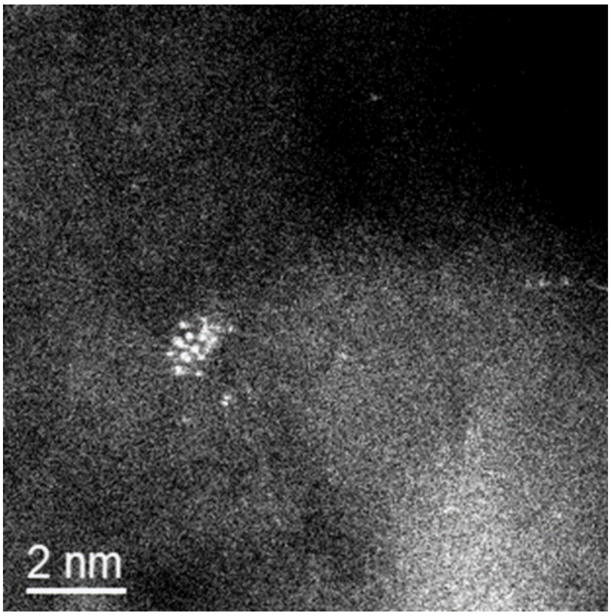

Supplementary Figure 9. AC-HAADF-STEM images. Images of Pt_n/ND@G after recycling test.

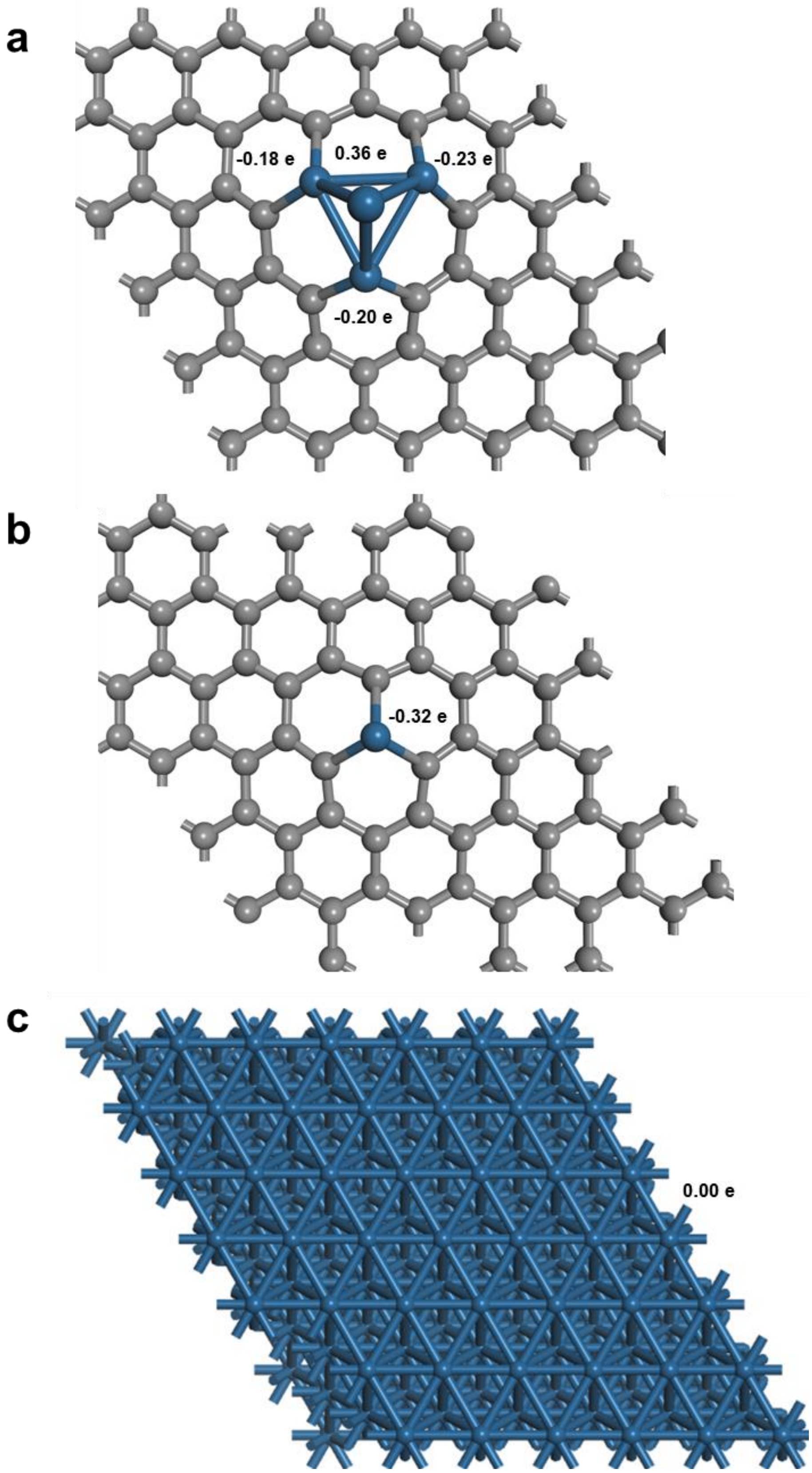

Supplementary Figure 10. Schematic structures and Bader charges. (**a**) Pt_4@Gr, (**b**) Pt_1@Gr, and (**c**) Pt(111).

Supplementary Table 1. Dispersion information of different Pt/ND@G samples.

Catalysts	Dispersion (%)
Pt_1/ND@G	100
Pt_n/ND@G	93
Pt_p/ND@G	66

Measured by H_2-O_2 titration method.

Supplementary Table 2. Curve-fit Parameters [a] for Pt L_3-edge EXAFS of Pt/ND@G catalysts.

**Catalysts**	**Paths**	***d*** **(Å)** [b]	**C. N.** [c]	**ΔE_0 (eV)**	**σ^2 (Å^2)** [d]
Pt_n/ND@G	Pt-O/C	2.00 ± 0.02	3.5 ± 0.3	9 ± 0	0.004 ± 0.002
	Pt-Pt	2.73 ± 0.02	2.5 ± 1.1		0.008 ± 0.004
Pt_1/ND@G	Pt-O/C	1.99 ± 0.01	1.7 ± 0.3	7 ± 1	0.005 ± 0.003
	Pt-Cl	2.31 ± 0.01	2.1 ± 0.7		0.005 ± 0.003

a: The data ranges used in the fit are $3.0 \leq k \leq 11.0$~13.0 Å^{-1} and $1.2 \leq R \leq 3.0$~3.5 Å, depend on the quality of data. S_0^2 was fixed at 0.732, obtained from the Pt foil measured at the same time. The number of variable parameters is out of a total of independent data points. *R*-factors for these fittings are all below 0.02. *b*: The half path length. The paths for Pt-O, Pt-Cl and Pt-Pt are from the crystal structure of PtO_2 ($P6_3mc$), K_2PtCl_6 (*Fm*-3*m*) and Pt (*Fm*-3*m*). *c*: average coordination number. *d*: Debye-Waller factor.

Supplementary Table 3. Comparison of the catalytic activity of Pt_n/ND@G with some reported catalysts toward 2,4-DNT hydrogenation.

Catalyst	Solvent	H_2 (MPa)	T (°C)	Time	Conversion (%)	Yield (%)	TOF (h^{-1})	Reference
Pt_n/ND@G	MeOH	1.0	25	2h	100	＞99	40647	This work
5% Pd /m-GO	MeOH	0.2	50	15min	82	82	1888	1
15Pt-ZrO_2-300	EtOH	1.0	80	20.8min	100	98.85	82161	2
60Pt-ZrO_2-300	EtOH	1.0	80	11.8min	100	98.42	-	2
20% Ni/HY	MeOH	2.59	75	2h	100	85	-	3
5% Pt/C	EtOH	1.0	50	10min	100	10	-	4
Pt/CrO_2	MeOH	2.0	60	-	＞98	＞99	-	5
Pd/$NiFe_2O_4$	MeOH	2.0	60	-	-	＞99	-	6
Ru/C	Isopropanol	8.3	110	4h	100	100	-	7
Fe-Co-Ir/C	MeOH	2.0	120	90min	-	99.5	-	8
Au/SiO_2	EtOH	4.0	140	4.5h	100	70.5	-	9
Co-L_1/C	THF	5	110	12h	-	83	-	10
CoS_2/PC	MeOH	3	110	6h	-	99	-	11

Supplementary Table 4. Pt actual loading of Pt_n/ND@G before and after reaction.

**Catalysts**	**Pt actual loading by ICP (%)**
Pt_n/ND@G before reaction	0.60
Pt_n/ND@G after reaction	0.57

Supplementary Table 5. The reaction energies *E*r (eV) of the hydrogenation of 2,4-DNT on Pt(111), Pt_4@Gr and Pt_1@Gr, respectively.

**Entry**	**E_r(2,4-DNT+3H_2(g) = 2A4NT +2H_2O(g))**	**E_r(2,4-DNT+6H_2(g) = 2,4-DAT+4H_2O(g))**
Pt(111)	−5.01 eV	−9.83 eV
Pt_4@Gr	−4.62 eV	−8.97 eV
Pt_1@Gr	−4.32 eV	−8.70 eV

Supplementary Table 6. The calculated reaction energies (*E*1/eV, *E*2/eV, *E*3/eV) of *R*1, *R*2, *R*3 on Pt(111), Pt_4@Gr, Pt_1@Gr catalysts with PBE+ZPE, PBE+D2+ZPE and PBE+D3+ZPE. *R*1, *R*2, and *R*3 is respectively represented the reaction steps of 2,4-DNT = 2,4-DNT*, H_2+2,4-DNT*= H_2*+2,4-DNT* and H_2*+2,4-DNT* = 2H*+2,4-DNT*.

	**PBE+ZPE**			**PBE+D2+ZPE**			**PBE+D3+ZPE**		
	*E*1	*E*2	*E*3	*E*1	*E*2	*E*3	*E*1	*E*2	*E*3
Pt(111)	−0.23	/	−1.13	−1.82	/	−1.19	−1.41	/	−1.30
Pt_4@Gr	−0.27	−0.16	−0.87	−1.31	−0.19	−0.75	−1.39	−0.10	−0.74
Pt_1@Gr	−0.53	0.01	0.18	−1.52	−0.30	0.50	−1.71	−0.28	0.47

Supplementary Table 7. The adsorption energies E_{ads} of intermediates on Pt(111), Pt_4@Gr and Pt_1@Gr, respectively.

**Species**	**Pt(111)**	**Pt_4@Gr**	**Pt_1@Gr**
2,4-DNT	−0.23 eV	−0.27 eV	−0.53 eV
2N4AT	−0.73 eV	−0.44 eV	−0.63 eV
2A4NT	−0.80 eV	−0.45 eV	−0.67 eV
2,4-DAT	−1.57 eV	−0.74 eV	−0.84 eV

Supplementary Table 8. The optimized structure, DFT energy (E_{DFT}) and corrected zero-point energy energy (E_{ZPE}) of 2,4-DNT, 2N4AT, 2A4NT, and 2,4-DAT on Pt (111).

Species	Structure	E_{DFT} (eV)	E_{ZPE} (eV)
2,4-DNT	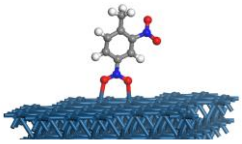	−956.84	3.48
2N4AT	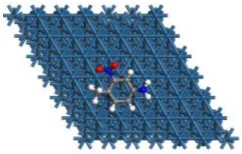	−954.29	3.98
2A4NT	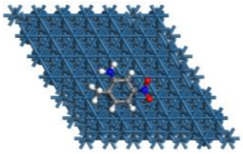	−954.46	3.92
2,4-DAT	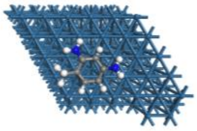	−951.89	4.36

Supplementary Table 9. The optimized structure, DFT energy (E_{DFT}) and corrected zero-point energy energy (E_{ZPE}) of 2,4-DNT, 2N4AT, 2A4NT, and 2,4-DAT on Pt_4@Gr.

Species	Structure	E_{DFT} (eV)	E_{ZPE} (eV)
2,4-DNT	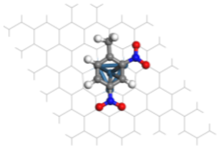	−762.75	3.49
2N4AT	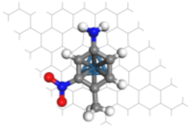	−759.80	3.91
2A4NT	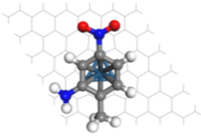	−759.95	3.90
2,4-DAT	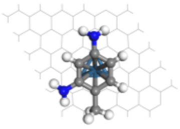	−756.87	4.32

Supplementary References

1. Salahshournia, H. & Ghiaci, M. Pd-Pt/modified GO as an efficient and selective heterogeneous catalyst for the reduction of nitroaromatic compounds to amino aromatic compounds by the hydrogen source. *Appl. Organomet. Chem.* **33**, e4832 (2019).
2. Ren, X., Li, J., Wang, S., Zhang, D. & Wang, Y. Preparation and catalytic performance of ZrO_2-supported Pt single-atom and cluster catalyst for hydrogenation of 2,4-dinitrotoluene to 2,4-toluenediamine. *J. Chem. Technol. Biotechnol.* **95**, 1675–1682 (2020).
3. Malyala, R. V. & Chaudhari, R. V. Hydrogenation of 2,4-Dinitrotoluene Using a Supported Ni Catalyst: Reaction Kinetics and Semibatch Slurry Reactor Modeling. *Ind. Eng. Chem. Res.* **38**, 906–915 (1999).
4. Zhao, F., Fujita, S., Sun, J., Ikushima, Y. & Arai, M. Hydrogenation of nitro compounds with supported platinum catalyst in supercritical carbon dioxide. *Catal. Today* **98**, 523–528 (2004).
5. Hajdu, V. *et al.* Precious-Metal-Decorated Chromium(IV) Oxide Nanowires as Efficient Catalysts for 2,4-Toluenediamine Synthesis. *Int. J. Mol. Sci.* **22**, 5945 (2021).
6. Hajdu, V. *et al.* Development of magnetic, ferrite supported palladium catalysts for 2,4-dinitrotoluene hydrogenation. *Mater. Today Chem.* **20**, 100470 (2021).
7. Oh, S. G. *et al.* One pot catalytic NO2 reduction, ring hydrogenation, and N-alkylation from nitroarenes to generate alicyclic amines using Ru/C-NaNO2. *Catal.*

Commun. **43**, 79–83 (2014).

8. Auer, E., Gross, M., Panster, P. & Takemoto, K. Supported iridium catalysts — a novel catalytic system for the synthesis of toluenediamine. *Catal. Today* **65**, 31–37 (2001).
9. Chen, Y., Qiu, J., Wang, X. & Xiu, J. Preparation and application of highly dispersed gold nanoparticles supported on silica for catalytic hydrogenation of aromatic nitro compounds. *J. Catal.* **242**, 227–230 (2006).
10. Westerhaus, F. A. *et al.* Heterogenized cobalt oxide catalysts for nitroarene reduction by pyrolysis of molecularly defined complexes. *Nat. Chem.* **5**, 537–543 (2013).
11. Wei, Z. *et al.* The synergic effects at the molecular level in CoS_2 for selective hydrogenation of nitroarenes. *Green Chem.* **20**, 671–679 (2018).
